# Grammatical Gender in Spoken Word Recognition in School-Age Spanish-English Bilingual Children

**DOI:** 10.3389/fpsyg.2022.788076

**Published:** 2022-02-17

**Authors:** Alisa Baron, Katrina Connell, Zenzi M. Griffin

**Affiliations:** ^1^Department of Communicative Disorders, University of Rhode Island, Kingston, RI, United States; ^2^Department of Speech, Language, and Hearing Sciences, University of Texas at Austin, Austin, TX, United States; ^3^Department of Spanish, Italian, and Portuguese, The Pennsylvania State University, State College, PA, United States; ^4^Department of Psychology, University of Texas at Austin, Austin, TX, United States

**Keywords:** grammatical gender, bilingual (Spanish/English), eye-tracking (ET), visual world paradigm (VWP), typically developing child

## Abstract

This study investigated grammatical gender processing in school-age Spanish-English bilingual children using a visual world paradigm with a 4-picture display where the target noun was heard with a gendered article that was either in a context where all distractor images were the same gender as the target noun (same gender; uninformative) or in a context where all distractor images were the opposite gender than the target noun (different gender; informative). We investigated 32 bilingual children (ages 5;6–8;6) who were exposed to Spanish since infancy and began learning English by school entry. Along with the eye-tracking experiment, all children participated in a standardized language assessment and told narratives in English and Spanish, and parents reported on their child's current Spanish language use. The differential proportion fixations to target (target − averaged distractor fixations) were analyzed in two time regions with linear mixed-effects models (LME). Results show that prior to the target word being spoken, these bilingual children did not use the gendered articles to actively anticipate upcoming nouns. In the subsequent time region (during the noun), it was shown that there are differences in the way they use feminine and masculine articles, with a lack of use of the masculine article and a potential facilitatory use of the feminine article for children who currently use more Spanish than English. This asymmetry in the use of gendered articles in processing is modulated by current Spanish language use and trends with results found for bilingual and second-language learning adults.

## Introduction

Both children and adults process speech incrementally, making use of what they have heard to anticipate the endings of words and sentences (e.g., Marslen-Wilson, 1987; Bates et al., 1996; Friederici and Jacobsen, 1999; Fernald et al., 2001). Even 2-year-olds can identify referents of familiar words with only partial word information (Fernald et al., 2001; Fernald and Hurtado, 2006). The present study examines incremental comprehension of spoken language in Spanish-English bilingual school-age children.

In many languages such as Spanish, nouns are assigned grammatical gender. For example, *the tomato* in French (*la tomate*) is feminine while in Spanish (*el tomate*) and Italian (*il pomodoro*) it is masculine. Learners use phonological, semantic, and morphological cues to assign nouns to gender classes (Karmiloff-Smith, 1979; Pérez-Pereira, 1991). In Spanish, definite articles are two of the most frequent words in Spanish. The log frequency for *el* is 4.50 and for *la* is 4.63 (with a maximum log frequency of 4.85 in EsPal, a Spanish Lexical Database; Duchon et al., 2013). Articles are almost always compulsory in a noun phrase, but as unstressed monosyllables, they have low saliency (Mariscal, 2009).

Monolingual children around 1;4–1;5 years of age produce bare nouns in Dutch, English, and German while those children learning Spanish and Italian tend to precede nouns with a “filler syllable” to hold the place for an article, or proto-article (Bottari et al., 1993; Peters and Menn, 1993; Lleó, 1998). Around 1;10, children acquiring a Romance language such as Spanish, produce a high percentage of articles and proto-articles and by 2;3 produce articles in an adult-like manner regardless of language. Thus, monolingual children make use of articles in spoken language comprehension from an early age. For example, Lew-Williams and Fernald (2007) found that 2- and 3-year-old's learning Spanish as their first language (L1) identified visual referents earlier in the context of different-gender articles (informative) than same-gender articles (uninformative). Specifically, children saw two pictures of objects with names of either the same [e.g., *la* pelota (fem.ball), *la* galleta (fem.cookie)], or different grammatical gender [*la* pelota, *el* zapato (masc.shoe)], as they heard a Spanish sentence referring to one of the objects. Children looked to the correct referent earlier on different-gender trials, when the article was potentially informative, than on same-gender trials, when the article could precede the name of either object. This study provided the first evidence that young Spanish-learning children with only 500 words in their expressive vocabularies already utilize morphosyntactic information in the process of establishing reference, exhibiting an anticipatory effect. Similarly, other researchers have shown children who speak other gendered languages also show sensitivity to gender early in development. By 25 months, French-learning children fixate referents earlier when preceded by informative gender-marked articles (van Heugten and Shi, 2009). However, at 24 months, Dutch-learning children are not yet sensitive to grammatical gender (van Heugten and Johnson, 2011), which may be due to the fact that they have yet to acquire the gender-marking system in Dutch, and articles are more obligatory in Spanish and French than Dutch. Additionally, there may be a difference in Romance language article acquisition vs. Dutch as Spanish has a more transparent gender system (typically -o ending for masculine nouns and -a ending for feminine nouns) (see for e.g., Pérez-Pereira, 1991; Sá-Leite et al., 2020). Although there are exceptions to the endings of masculine and feminine nouns, and there are opaque endings as well (-e ending), overall, gender acquisition and processing is facilitated by these regularities (Sá-Leite et al., 2019). Dutch, on the other hand, has an opaque gender system where grammatical gender values are either “common” or “neuter” (Sá-Leite et al., 2019). For example, in Dutch, “de fiets [the bicycle]” is common and “het huis [the house]” is neuter. Therefore, due to the lack of transparency and regularity in Dutch, children may take longer to acquire the grammatical gender system.

While the development of the use of gendered articles in children is under investigation, it has been shown repeatedly that adult monolingual speakers can make use of such cues to facilitate processing. Lew-Williams and Fernald (2007) for example, tested children in the study described earlier, and also included a group of monolingual Spanish-speaking adults. Their results showed that these adults were able to identify the correct referent faster when gender was informative compared to when it was not. They were also able to do so faster than the children in the study. This result of monolingual adult speakers using informative gender to facilitate online processing has been replicated several times in multiple L1s, including Italian, French, and Russian (e.g., Bates et al., 1996; Akhutina et al., 1999; Dahan et al., 2000; Dussias et al., 2013).

Although monolingual speakers have been overwhelmingly shown to be able to use gender-marked articles to identify familiar referents, adults learning a second language (L2) with gender-marking show varied success in using gender-marked articles in online processing. Grammatical gender appears to be one of the more difficult aspects of language for L2 learners to master (Carroll, 1989). Replicating their earlier work with monolingual adults and children, Lew-Williams and Fernald (2010) tested adult L2 learners of Spanish with about 5 years of Spanish classroom learning. The learners attended to the correct referent with equal speed, regardless of whether the articles were informative or not, suggesting that they were unable to use gender as a cue to facilitate online processing. Even when frequency of exposure to article-noun pairs was controlled by training adults on novel nouns, native Spanish speakers fixated referents earlier when grammatical gender was informative whereas L2 learners did not (Lew-Williams and Fernald, 2010). Counter to these results, Dussias et al. (2013) found that English-speaking learners of Spanish were able to use gender to facilitate the processing of an upcoming word, but this ability was modulated by proficiency. So, while it remains unclear if L2 learners are reliably able to use gender as a cue to facilitate processing, it seems that proficiency may likely play an important role (Dussias et al., 2013; Hopp, 2016; Hopp and Lemmerth, 2018).

For more balanced bilinguals, several studies have shown that, like their monolingual counterparts, they are able to use grammatical gender to facilitate processing in different-gender contexts (informative) compared to same-gender contexts (uninformative), however, an asymmetry arises in the use of the masculine and feminine articles. Many researchers have discussed and explained masculine default accounts. Harris (1991) posited that the masculine gender in Spanish is the unmarked or default gender as there are numerous Spanish examples that corroborate this argument. He further claims that the masculine gender is the “absence of any information about gender in lexical entries” (p. 44). Others have also proposed the masculine default gender in French (Hulk and Tellier, 1999), in Italian (Riente, 2003), in Greek (Tsimpli and Hulk, 2013), among others. Hur et al. (2020) also noted that agreement in the feminine gender appears to be more salient or more recognizable in both online and offline receptive tasks when compared to the “unmarked default status” of the masculine gender (Domínguez et al., 1999; Smith et al., 2003; Alemán Bañón and Rothman, 2016; Beatty-Martínez and Dussias, 2019). As there is a consensus that masculine appears to be the default, feminine thus seems to be the marked option.

Spanish-English speaking adults have been shown to use the feminine article to facilitate processing but show no use of the masculine article. This gender asymmetry has been shown for Spanish-English bilinguals from Latin-America (Valdés Kroff et al., 2017) as well as Italian-Spanish learners from Italy (Dussias et al., 2013). Valdés Kroff et al. (2017) explain this effect by saying since *el*, the masculine article, is used extensively as the default article in code-switching, this may lead bilinguals to ignore it as a cue when preceding a noun during comprehension. Additionally, De la Cruz Cabanillas et al. (2007), found that 82% of gendered loanwords in their corpus were masculine, further giving rise to the default status of the masculine gender. If the masculine gender is indeed the default or unmarked gender, then it stands to reason that it is ignored in terms of facilitatory processing, and the non-default (or marked) feminine article is therefore informative enough to cue facilitatory processing. Thus, there appears to be an underlying difference in the representation and processing of masculine and feminine gender in Spanish due to distributional asymmetries between them, which leads to biases in gender assignment (Beatty-Martínez and Dussias, 2019).

This account of the gender asymmetry effect is strengthened by complementary results outside the realm of gender. Connell et al. (2021) tested L1-Spanish L2-English learners for their ability to anticipate an upcoming word based on the form of the indefinite article in English, with “a” being used before consonant initial words and “an” before vowel initial words. Their results showed that L2-English learners were able to use the phonological form of an article to anticipate the upcoming word, but that they only did so when the article was “an” and did not use the “a” article to cue anticipatory processing. In this case, “anticipatory” processing is used as opposed to “facilitatory” since the effects were found before the onset of the target noun. For the remainder of the paper, “anticipatory” will be used to denote processing that occurs strictly before a target word is spoken, and “facilitatory” will be used to refer to processing advantages including, but not limited to, the target word itself.

This ability to use the feminine article was further modulated by proficiency, with high-proficiency learners using the “an” to anticipate to a greater degree than the low-proficiency learners. While not a gender distinction, the alternating forms of the English indefinite article do exhibit a similar status asymmetry, with the “a” form arguably serving as the default form, and this asymmetry is reflected in online processing as is with grammatical gender.

In summary, monolingual toddlers and adults can use gender-marked articles to facilitate spoken word recognition. Bilinguals can also use gender-marked articles to facilitate spoken word recognition, however, proficiency appears to play a role for late learners and there seems to be a difference in the way masculine and feminine genders are processed.

Behaviorally, we know that children with language disorders are less accurate in producing gender-marked articles than their typically-developing peers (e.g., Morgan et al., 2013). This suggests that they might also be less likely to comprehend articles compared to their peers. Initially, we planned to test whether bilingual children with language disorders were less likely to use different gender-marked articles (informative) to speed word recognition than bilingual typically-developing children (the control group) were. Like Lew-Williams and Fernald's (2007) younger native Spanish-speaking monolinguals, we expected older bilingual children to fixate referents more rapidly in contexts where articles were informative rather than uninformative. However, preliminary tests for gender sensitivity in our sample of typically-developing bilingual children showed no difference, despite the fact that they were enrolled in dual language (English-Spanish) schools. Rather than continue to recruit children with language disorders, the focus of the study turned to typically-developing bilingual children to evaluate the factors leading to their different gender processing from that of younger monolinguals. Here we report the analyses from this deviation from our planned study and discuss implications for understanding neurotypical bilingual language development.

In order to investigate how typically-developing Spanish-English bilingual children comprehend and attend to gender-marked articles in Spanish, a visual world paradigm was used to examine gender-marked articles in informative and uninformative contexts. Children also completed a narrative task to elicit spontaneous production of gender-marked articles.

The following research questions were addressed:

1. Do Spanish-English bilingual children take advantage of informative grammatical gender marking on articles in Spanish in anticipatory or facilitatory processing?
2. Do bilingual children show a differential use of the gendered articles by masculine or feminine like that shown by bilingual adults?
3. Does current Spanish use (input/output) influence bilingual children's ability to use gendered articles in an anticipatory or facilitatory manner?

## Materials and Methods

### Participants

Fifty-one children between the ages of 5;6-8;6 were recruited from 4 dual language elementary schools in Austin, Texas. All parents and children gave informed consent/assent to participate in the study and were compensated for their participation. This study was approved by the Institutional Review Board at the University of Texas at Austin. Three participants were excluded due to language impairment, 5 due to frequent track loss and inability to complete a 9-point validation, 3 due to lack of fixations in either condition in the analysis time window (which can arise from using peripheral vision, looking off screen, etc.), 1 due to computer error, 4 due to English as a first language, and 3 due to no Spanish behavioral data. Thus, analyses are based on data from 32 children (14 F).

Children were categorized as typically-developing if they scored within normal limits on the Bilingual English Spanish Assessment (BESA; Peña et al., 2018; ages 3–6;11) or the Middle Extension (BESA-ME; Peña et al., 2008; ages 7–9;11) and no parent or clinician concern was noted (Gutiérrez-Clellen and Simón-Cereijido, 2007). These tests are used to assess language ability in bilingual children in both English and Spanish. A certified bilingual speech-language pathologist (first author) administered and scored all tests.

Parents completed the Bilingual Input Output Survey (BIOS; Peña et al., 2018) in which they provide information on the child's language use since birth and their child's current language input (how much they hear) and output (how much they speak) on an hourly day-to-day basis at home. Teachers reported spending equal amounts of classroom time speaking English and Spanish. As the correlation between input and output within languages was 0.91, input and output data within each language were averaged for all analyses. This variable is called Spanish Use. Table 1 shows participant means for age, age of first exposure to English and Spanish, mother education based on Hollingshead (1975) index (a proxy for socioeconomic status), and Spanish Use at the time of testing. All children were exposed to Spanish from birth and, on average, heard and spoke more Spanish than English at the time of testing.

**Table 1:** Participant characteristics presented in means and standard deviations.

**Characteristic**	**M**	**SD**
Age (months)	86.60	(10.20)
SES/Mother's Hollingshead Index	3.50	(1.84)
Age of first exposure to English (years)	2.82	(1.98)
Age of first exposure to Spanish (years)	0	(0)
Spanish input and output (percent)	60.40	(21.90)

### Materials

**Figure 1:**
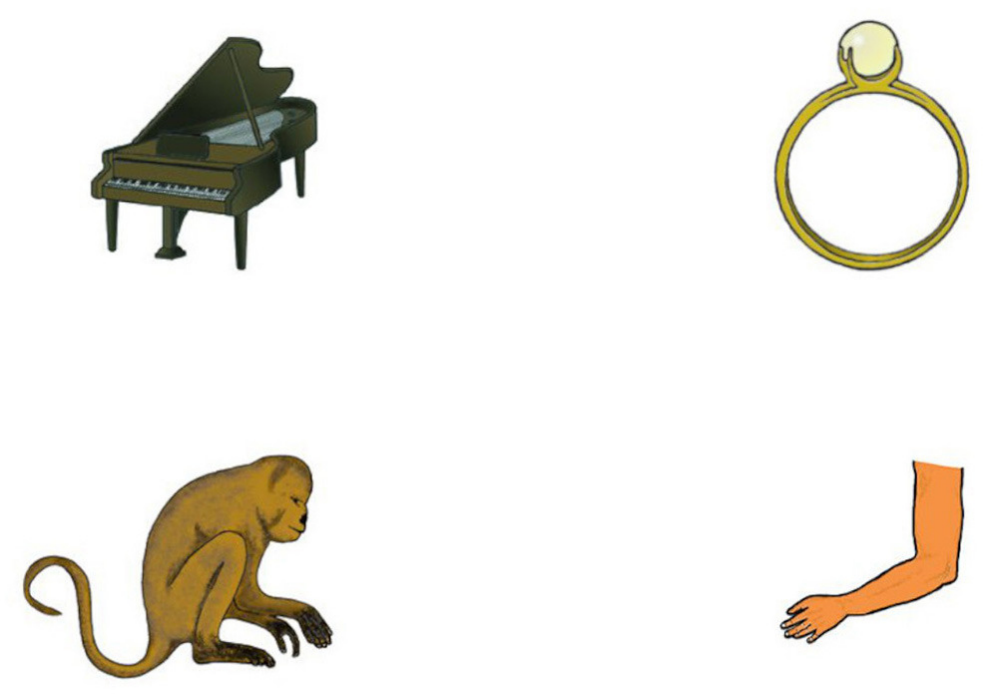
An example of a same-gender condition display, including el brazo [masc.arm], el piano [masc.piano], el anillo [masc.ring], and el mono [masc.monkey].

Thirty familiar objects were selected to be targets on experimental trials. Half of the target names had masculine grammatical gender and half feminine. Twenty-two of the thirty nouns (73%) had a transparent gender (words ended in -o or -a) while eight had an opaque gender (words ended in -n, -j, -z, -e, -r). Of the eight opaque words, 7 were masculine and 1 was feminine. Each target was combined with three unrelated distractors with the same gender as the target in the same-gender condition and 3 unrelated distractors of the opposite gender as the target object in the different-gender condition. Using EsPal (Duchon et al., 2013), target objects were found to be of equal log frequency by gender (*p* = 0.88^1^). Distractors were not phonological competitors of the target in that they did not match in consonant-vowel onset and did not rhyme. The distractor objects did not differ from target objects in log frequency (*p* = 0.81; see Footnote 1), in word length (*p* = 0.63), familiarity (*p* = 0.94^2^), imageability (*p* = 0.80^3^), or concreteness (*p* = 0.48^4^). Stimuli were colored Snodgrass and Vanderwart line drawings (Rossion and Pourtois, 2004) depicting animals, body parts, clothes, household items, foods, vehicles, instruments, toys, and other objects young children are familiar with. The Snodgrass and Vanderwart pictures were standardized for Spanish (Sanfeliu and Fernandez, 1996) and distractor objects did not differ from target objects in familiarity (*p* = 0.78), visual complexity (*p* = 0.06), or naming agreement (*p* = 0.87; see Footnote 1). The target objects were slightly less visually complex than the distractors, although the difference was not significant. Each object occupied a distinct quadrant of the display screen (example display Figure 1). Target objects occurred in each quadrant equally^5^ often to discourage anticipation of their positions. The pictures were edited to fit within 250 × 250 pixels.

A female Spanish-English bilingual speaker was recorded saying “*enséñame [show me]*” and the appropriate definite article with each target noun^6^. She spoke slowly to minimize co-articulation in order to make grammatical gender the only potentially useful source of information about the upcoming noun and to have consistent timing for the onset of information. Recordings were edited to extract one token each of *enséñame*, the masculine article *el*, and the feminine article *la*. Similarly, target nouns were spliced out of the recordings and saved as their own sound files. The mean target noun duration was 669 ms (SD = 128 ms).

#### Design and Procedure

All participants were presented with the same three practice trials initially (2 same-gender and 1 different-gender contexts). Then thirty experimental trials were presented in a pseudo-random order with the constraint that the same condition did not appear more than three times in a row. Two lists of stimuli were constructed so that each participant saw every target object once, half in each condition, with the assignment of target to condition counterbalanced across lists and thereby participants. Seventy-five of the ninety distractors (83%) appeared in both conditions across the two lists, so their properties were roughly counterbalanced.

Eye movements were tracked with an SR Research EyeLink 1000, sampling at 1,000 Hz. Eye-tracking began with a 9-point calibration and validation routine. Participants were instructed to listen carefully and look at what each sentence described. Each trial began with a central validation point followed by a 200 ms preview of the objects. A central fixation cross appeared for the duration of the sound file *enséñame*. Approximately 550 ms later, an article sound file began, *el* (365 ms) or *la* (300 ms), and finally after a pause of ~370 ms, the target noun began. As the youngest children in the study were 5;6, a positive non-verbal reinforcement was added (experimenter offered a thumbs up or quiet clapping) to motivate them to continue. After participants fixated on the target object for 500 ms, a red square appeared around the target for 300 ms, and the trial ended.

After the eye-tracking task, participants were introduced to a wordless picture book and were asked to tell a story in each language: *Frog Goes to Dinner* (Mayer, 1974) and *Frog On His Own* (Mayer, 1973). When comparing groups of children who told these two stories, negligible differences have been observed in language measures (Heilmann et al., 2016). Both the book and language order were counterbalanced across participants. Stories were transcribed and coded for mean length of utterance in words (MLUw), number of different words (NDW), and percentage of grammatical sentences based on the procedures described by Miller and Iglesias (2008) using Systematic Analysis of Language Transcripts (SALT) software. Inter-scorer reliability was 96.2% at the word level and 88.7% for the grammaticality of the sentence.

## Results

Language measures (MLUw, NDW, and grammaticality) mean values for English and Spanish are shown in Table 2. MLUw was slightly higher in English while NDW was similar across both languages and grammaticality was higher in Spanish. Pearson correlations between children's ages, language history, and measures of language skills are shown in Table 3.

**Table 2:** Language measures presented in means and standard deviations.

**Language measure**	**English**		**Spanish**	
	**M**	**SD**	**M**	**SD**
Mean Length of Utterance in Words	8.47	(2.28)	6.85	(1.59)
Number of Different Words	88.70	(30.20)	86.10	(21.6)
Percentage of Grammatical Utterances	53.02	(20.90)	72.20	(22.90)

**Table 3:** Pearson correlations between participant age, language history, and measures of language skills.

	**Spanish Input/Output**	**English 1st exposure**	**Spanish MLUw**	**Spanish NDW**	**Spanish GRAM**	**Article accuracy**
Age	−0.21	−0.09	0.56^***^	0.40^*^	−0.18	−0.08
Spanish input/output		0.040^*^	0.41^*^	0.01	0.47^**^	0.39^*^
Eng 1st exp			−0.02	0.05	−0.05	0.11
Spanish MLUw				0.045^**^	−0.19	0.13
Spanish NDW					−0.23	0.0
Spanish GRAM						0.61^***^

*MLUw, Mean length of utterance in words; NDW, Number of different words; GRAM, grammaticality*.

* *p < 0.05*;

** *p < 0.01*;

*** *p < 0.001*.

Spanish-dominant bilingual children in the US typically acquire articles between 5;0 and 6;10, which is later than most monolinguals (e.g., Pérez-Pereira, 1989; Gutiérrez-Clellen et al., 2006; Morgan et al., 2013). At this age, monolingual children are 100% accurate when using grammatical morphemes in everyday conversations. Our sample of children produced gender-marked articles with 85.4% (SD = 25) accuracy in the elicited production portion of the BESA/BESAME (3–4 items). When telling stories, children produced articles with 89.2% (SD = 21.4) accuracy. A grammatical morpheme is typically considered “acquired” when a child uses the structure accurately at least 80% of the time in obligatory contexts (i.e., Bloom and Lahey, 1978). These accuracy levels suggest that most of the children have acquired gender-marked articles and that their accuracy is typical of Spanish-dominant bilinguals of the same age.

**Figure 2:**
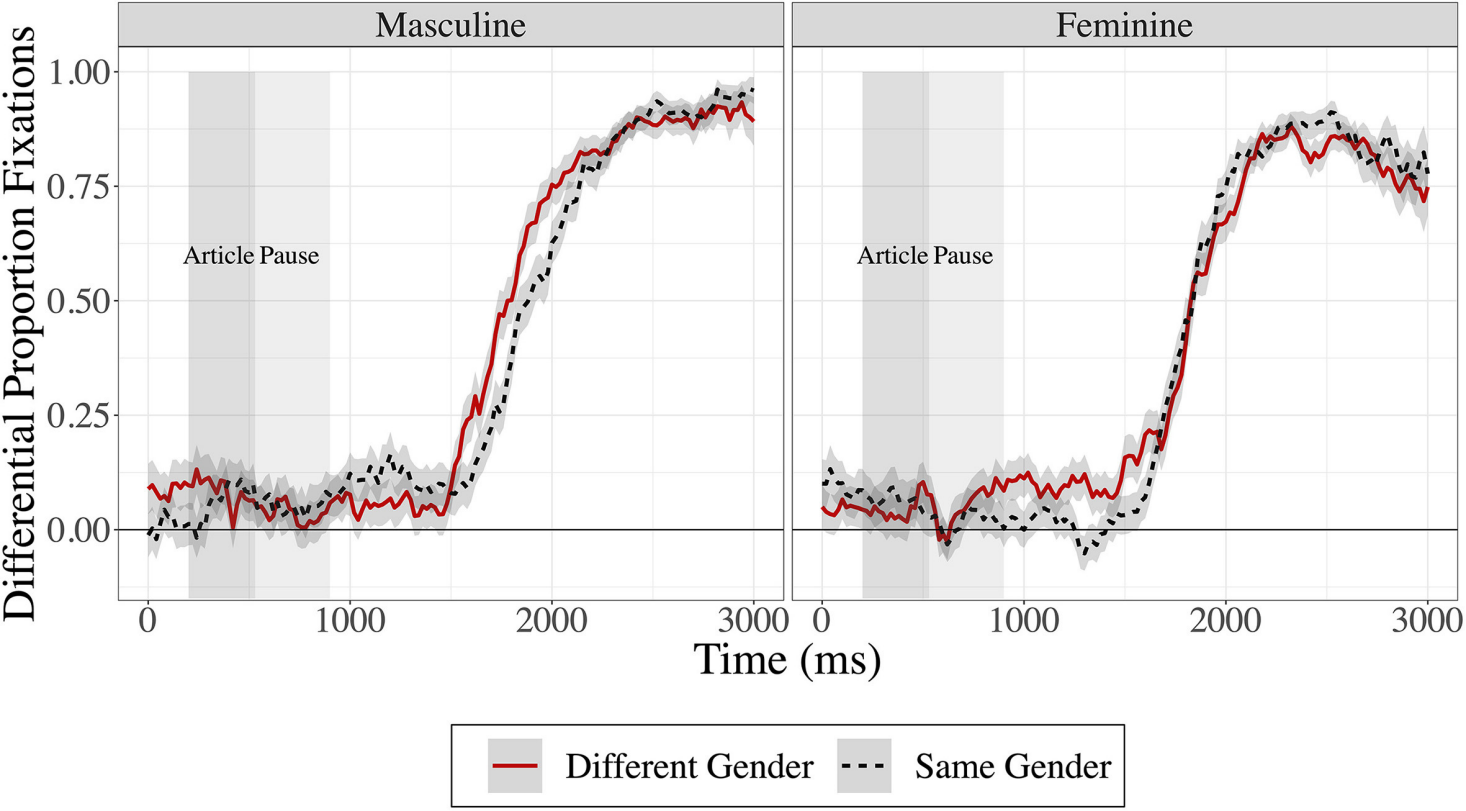
Differential proportion fixations to target, with fixations in the different-gender condition in solid red lines and fixations in the same-gender condition in dashed black lines. Masculine targets are presented in the left panel and feminine targets are presented in the right panel. Time in milliseconds is presented on the x-axis and differential proportion of fixations to target is presented on the y-axis. The shaded regions represent ±1 standard error of the mean.

The eye-tracking data was exported using SR Research Data Viewer software. An interest period was set from the beginning of the article until the participant fixated the correct target for 500 ms or more. A Time Course (Binning) report was used to export the data. This report binned time into 20 ms bins and excluded samples that fell outside of four pre-defined interest areas around the images and samples during blinks or saccades. Trials for which the target object had never been correctly fixated were excluded from the eye movement analyses (5.1%). All further analyses were conducted in *R* (R Core Team, 2013). Further data cleaning in R included excluding trials for which the target object had not been correctly fixated on within 5,000 ms (11.4%). The fixations were time locked to the onset of the article preceding the target noun and included a 200 ms baseline (for the time it takes to plan and launch a saccade; Hallett, 1986). Differential proportions of fixations to target (DPFT) were then calculated for use in the analysis by subtracting the averaged proportions of distractor fixations from the proportions of target fixations. The DPFT are presented in Figure 2 below.

**Figure 3:**
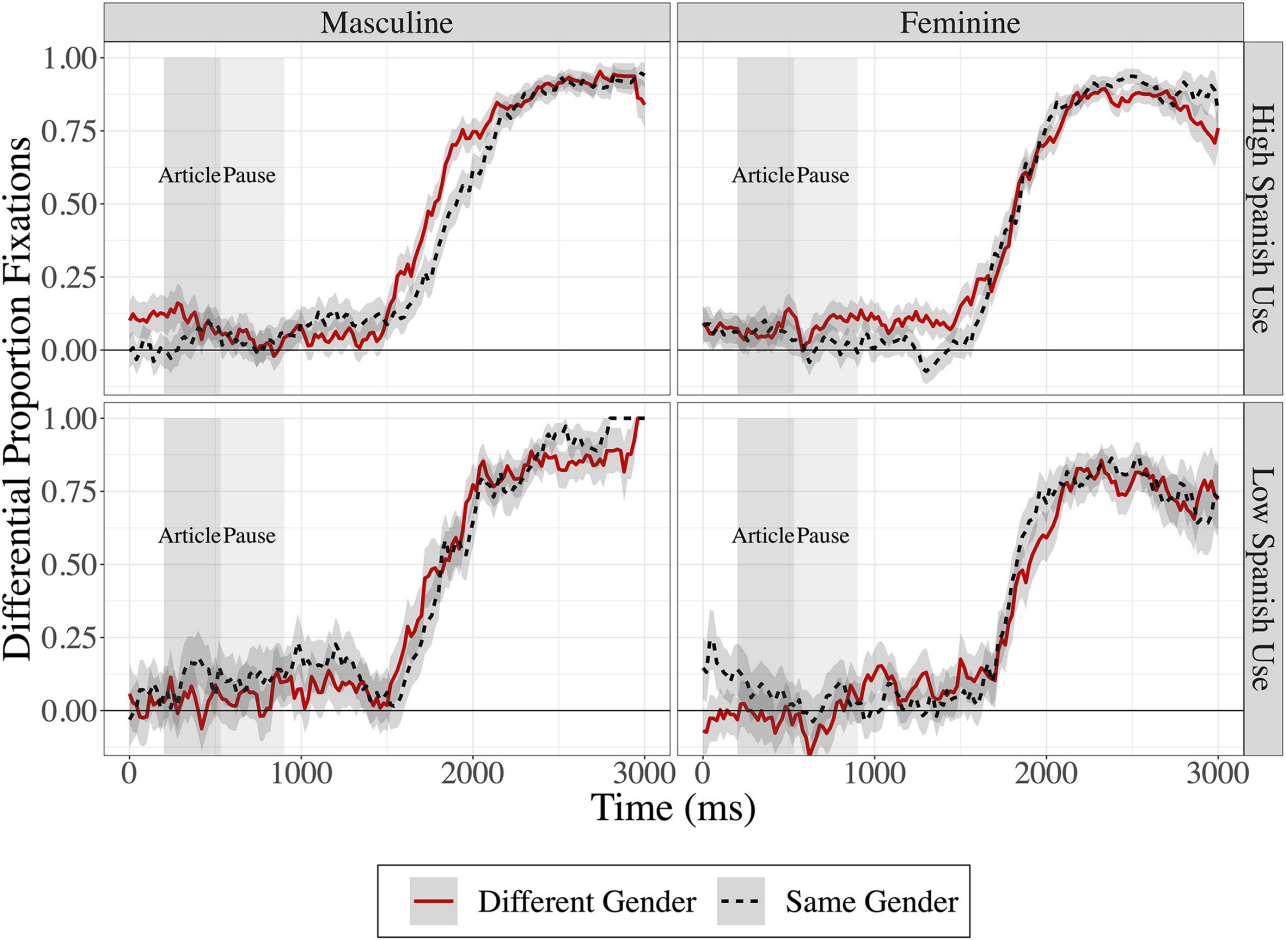
Differential proportion fixations to target, with fixations in the different-gender condition in solid red lines and fixations in the same-gender condition in dashed black lines. Masculine targets are presented in the left panel and feminine targets are presented in the right panel. Participants with ≥50% Spanish Use are shown in the top row, and participants with <50% Spanish Use are presented in the bottom row. Time in milliseconds is presented on the x-axis and differential proportion of fixations to target is presented on the y-axis. The shaded regions represent ±1 standard error of the mean.

In Figure 2, data points below 0 reflect that participants were looking at the distractors more than the target; points at 0 reflect equal proportion fixations to target and distractors; and points above 0 reflect that participants were looking more at the target than the distractors. Figure 2 illustrates fixations on the target object in the context of same- (black dashed) and different-gender (red solid) conditions with masculine-target trials presented on the left and feminine-target trials presented on the right. Figure 3 shows these same results separated by a split of the participants' reported combined Spanish Use (language input and output) with high Spanish use being those with over 50% use of Spanish and low being <50% use of Spanish.

### Lexical Anticipation

To investigate effects of lexical anticipation, the DPFT were analyzed with a linear mixed-effects model (LME) using the Buildmer (Voeten, 2020) package in R on a window from 530 to 900 ms, which includes fixations in the pause region after the article had been heard, but before the onset of the target noun. The Buildmer function in the Buildmer is provided with a maximal model including all interactions and random effects justified by the design and performs a stepwise and elimination of effects with forward and backward effect-selection based on the change in log-likelihood ratio tests of compared models. The model given to the Buildmer function for this analysis included fixed effects of condition (same vs. different article), target gender (feminine vs. masculine) and Spanish Use (input/output) (high vs. low Spanish Use). The model also included random intercepts of participant and item, random slopes of condition and target gender for participant, and random slopes of Spanish Use for item. The same-article masculine condition served as the baseline to which all comparisons were made. The model output by the Buildmer function as the maximal model included fixed effects of condition, target gender, and Spanish Use and the interaction of target gender and Spanish Use as well as all random effects. The low Spanish Use, masculine trials across both conditions serve as the baseline to which all comparisons are made.

Table 4 summarizes the results of the maximal model. The significant effect of target gender [ß = −0.27, *t*_(62.9)_ = −2.80, *p* = 0.008] indicates that for low Spanish Use participants, there were significantly fewer looks to the feminine items compared to masculine items, regardless of condition. The significant interaction between target gender and Spanish Use [ß = −0.26, *t*_(59.30)_ = −2.22, *p* = 0.030] indicates that the effect of gender seen for the low Spanish Use participants reverses directionality, with high Spanish Use participants looking at the feminine items more than the masculine items. It is important to note here that the effect of condition was not significant, and no interaction with this effect significantly improved the fit of the model (as it was not included by the Buildmer function in the final model), indicating that the condition of the trial (same- or different-gender) did not significantly improve the model.

**Table 4:** Results of LME on DFPT by target gender, condition, and Spanish use in the pause region.

	**Estimate**	**Std. error**	**df**	***t-*value**	**Pr(>|t|)**
Intercept	0.15	0.08	78.13	1.84	0.069
Condition	0.02	0.06	71.24	0.38	0.704
Gender	−0.28	0.10	62.85	−2.76	0.008
Spanish use	−0.10	0.09	60.19	−1.16	0.252
Spanish use: Gender	0.26	0.12	59.30	2.22	0.030

### Lexical Facilitation

The results just presented speak to processing during the pause after the article has been spoken, but before the noun, and can thus reflect anticipatory processing. Inspection of Figure 3 reveals that looks to the correct target do not begin to drastically increase until well into the word in all conditions, at least 500 ms after the end of the article. In order to look at the effects of gender on the processing of the spoken word itself, here, we might expect to find carry-over effects or effects of facilitation during the processing of the word itself. To investigate these effects, the same analysis described above was conducted on the period of time after the pause during the spoken word. This window began at the end of the pause (i.e., the beginning of the target word) and extended to 1,500 ms. All details of the model were identical to the main model with the only change being the time window of the analysis. The maximal model included all fixed effects and all possible interactions. The low Spanish Use, same-gender, masculine target trials serve as the baseline to which all comparisons are made.

Table 5 summarizes the results of the post-pause analysis window. The significant interaction between target gender and condition [ß = −0.30, *t*_(72.1)_ = −2.7, *p* = 0.03] indicates that the (non-significant, negative) effect of gender becomes more negative from same- to different-article trials. In other words, there was a greater reduction in looks to the feminine items from same gender trials to different gender trials in low Spanish Use participants compared to the reduction in looks in the masculine items. The significant 3-way interaction [ß = 0.34, *t*_(66.29)_ = 2.35, *p* = 0.022] indicates that the previously described effect of target gender and condition reverses direction from low Spanish Use to high Spanish Use. This means that what was a reduction in looks to the feminine article items between same- and different-gender trials is reduced (and in fact reverses directionality, from low to high Spanish Use), and this reversal indicates that high Spanish Use participants show a greater positive increase in looks to target in the different gender items for feminine targets compared to masculine targets.

**Table 5:** Results of LME on DFPT by target gender, condition, and Spanish use in the post-pause region.

	**Estimate**	**Std. error**	**df**	***t*-value**	**Pr(>|t|)**
Intercept	0.08	0.10	73.07	0.83	0.407
Gender	−0.06	0.14	73.92	−0.42	0.679
Condition	0.08	0.12	75.41	0.67	0.502
Spanish use	0.02	0.10	70.24	0.15	0.878
Gender: Condition	−0.3	0.14	72.10	−2.27	0.026
Gender: Spanish use	−0.05	0.14	71.33	−0.33	0.742
Condition: Spanish use	−0.06	0.13	74.24	−0.46	0.645
Gender: Condition: Spanish use	0.34	0.14	66.30	2.35	0.022

## Discussion

The purpose of this study was to investigate grammatical gender processing in school-age Spanish-English bilingual children. Past work has focused on toddlers and adults who are monolingual, bilingual, or second language (L2) learners. Here, however, we hone in on early school-aged bilingual children who have been exposed to Spanish since infancy and have had varied experiences with English.

The first question posited was whether or not Spanish-English bilingual children can take advantage of grammatical gender marking on articles in online processing. The second question asked was if these bilingual children would show a similar gender asymmetry effect as seen in bilingual adults. Lastly, the third question asked was if current Spanish use (input/output) influences bilingual children's ability to use gendered articles in an anticipatory or facilitatory manner. We addressed these three questions in anticipatory and facilitatory processing by looking at two separate time regions (pause between article and noun and during the word).

The results of the present study showed that this group of bilingual children do not take advantage of informative gender marking on articles to actively anticipate an upcoming word. During the pause region, these bilingual children did not look at the correct target item more when the target item differed in gender from the distractors (informative) compared to when the gender of the target matched that of the surrounding distractors (uninformative). This demonstrates that these bilingual children did not use grammatical gender of the article to anticipate upcoming information in the speech signal.

The second analysis looked at effects of lexical facilitation and was conducted on the period of time while the target word was being spoken,. The three-way interaction of target gender, condition, and Spanish Use (as shown in Table 5) indicates that the use of feminine and masculine gender as cues for processing is different depending on children's current Spanish Use. Children who currently use Spanish less than English, appear not to be using the gendered article as a cue at all. Even more so, in the feminine target trials, they seem to use the masculine default as a preference (Otheguy and Lapidus, 2003; Balam, 2016) and look at the three masculine distractors more than the feminine target since as discussed previously, masculine is considered the default gender (see for example: Harris, 1991; Valdés Kroff et al., 2017; Balam et al., 2021). As the masculine is the default or unmarked gender, it may thus be easier to acquire and use (Pérez-Pereira, 1991). In other words, in the absence of using an article cue, these children seem to be anticipating the more frequent gender, which is masculine. On the other hand, children who use Spanish more than English showed a significant increase in looks to the feminine objects compared to those children who use Spanish less. These bilingual children who speak Spanish more than English, do not show the same masculine preference and in fact, may even use the feminine article to facilitate processing.

Furthermore, it was shown that bilingual children do not use the feminine and masculine articles in the same way in processing. This lack of use of the masculine article and potential use of the feminine article by bilingual children who speak more Spanish demonstrates an asymmetry. This asymmetry in the use of the masculine and feminine genders in processing is trending with results found with bilingual and second-language learning adults (Dussias et al., 2013; Valdés Kroff et al., 2017) and is discussed extensively in regards to a masculine gender default (see for example Harris, 1991; Hur et al., 2020). Collectively, this gender asymmetry in processing has been found for Spanish-English adult bilinguals, adult Italian learners of Spanish, and now the present work suggests that these findings may be relevant to school-aged Spanish-English bilingual children. Adding in the evidence for a parallel asymmetry shown for English learners in the use of “a” vs. “an,” these results may suggest that the root of this asymmetry is not only restricted to simply code-switching or attrition accounts as previously posited and may indeed be more related to bilingualism and current language use in general (De la Cruz Cabanillas et al., 2007; Valdés Kroff et al., 2017).

### Limitations

In looking-while-listening and visual world paradigms, participants are often asked to name stimulus objects prior to the experiment, or they hear a label for each object (Dahan et al., 2000; Lew-Williams and Fernald, 2007, 2010). Thus, the objects, their target names, and grammatical gender are typically primed prior to experiments. In this study, we did not pre-expose participants to objects or their names. As a result, we cannot be certain that the children would have consistently provided the same label as we used.

The majority of the target nouns had transparent gender while 26% had opaque gender (87% of which were masculine gender). It is possible that bilingual children had to spend more cognitive resources processing these opaque, masculine nouns, resulting in the lack of anticipatory online processing. This is in line with previous literature which has shown that opaque nouns in Spanish require more effortful processing than transparent nouns (Hernandez et al., 2004). The percentage of opaque gender nouns is slightly higher than Dussias et al. (2013), who also noted that cognitive processing may be more effortful for opaque nouns which potentially led to a lack of an anticipatory effect for low-proficiency Italian-Spanish adult learners in the masculine different-gender trials. However, in this study, no anticipatory effect was noted in either masculine or feminine articles even though there was only one feminine opaque gender noun. An additional potential limitation is that accuracy was calculated based on eye movements rather than a verbal response or overtly clicking the image of their choice. Lastly, given the variability inherent in data collected with children, the small sample size may have influenced our ability to detect anticipatory processing in these bilingual children.

### Conclusion and Future Directions

In sum, these school-aged Spanish-English bilingual children did not demonstrate the ability to use grammatical gender in Spanish anticipatory online processing. However, an asymmetry between the use of masculine and feminine articles was seen while children were hearing the noun and indicates that the amount of current Spanish use may differentially influence how gendered articles are used to facilitate processing. This result is similar to bilingual adults asymmetrical use of gender. Other factors may modulate the ability for school-age children to utilize this gender cue in a facilitatory or anticipatory way. Additionally, to the authors' knowledge, this is the first grammatical gender eye-tracking study focusing on school-aged children. As this is an important age for language development, acquisition, and/or attrition, further research on grammatical language processing is needed for this age group. Future work may want to directly compare bilingual and monolingual children with bilingual and monolingual adults to further clarify the nature of the gender asymmetry in these groups.

## Data Availability Statement

The raw data supporting the conclusions of this article will be made available by the authors, without undue reservation.

## Ethics Statement

The studies involving human participants were reviewed and approved by University of Texas at Austin Institutional Review Board. Written informed consent to participate in this study was provided by the participants' legal guardian/next of kin.

## Author Contributions

AB and ZG contributed to the conception and design of the study. AB collected all of the participant data, wrote the first draft of the manuscript, and wrote sections of the final manuscript. KC performed the statistical analyses and wrote sections of the final manuscript. All authors contributed to manuscript revision.

## Funding

The Texas Speech Language and Hearing Association provided the first author with funding through the Elisabeth Wiig Doctoral Student Research Fund which was used to pay participants who were included in the study presented in this article.

## Conflict of Interest

The authors declare that the research was conducted in the absence of any commercial or financial relationships that could be construed as a potential conflict of interest.

## Publisher's Note

All claims expressed in this article are solely those of the authors and do not necessarily represent those of their affiliated organizations, or those of the publisher, the editors and the reviewers. Any product that may be evaluated in this article, or claim that may be made by its manufacturer, is not guaranteed or endorsed by the publisher.

## Footnotes

^1^One target object was excluded from this, as no frequency data was available in the database.

^2^Eight target objects and 19 distractors were excluded as no familiarity data was available in the database.

^3^Eleven target objects and 22 distractors were excluded as no imageability data was available in the database.

^4^Eight target objects and 18 distractors were excluded as no concreteness data was available in the database.

^5^Target objects occurred in one quadrant one additional time compared to the rest of the three quadrants.

^6^When a masculine noun follows *mira a [look at]*, the definite article *el* combines with *a* and becomes the contraction *al*. This does not occur with feminine nouns. Thus, *enséñame* was used instead.
